# Lithium-Induced Sialorrhea

**DOI:** 10.7759/cureus.38370

**Published:** 2023-05-01

**Authors:** Bamidele O Johnson, Oluwaseun Oke, Christian Nwabueze, Muhammad Azam, Christianah Y Ogunlesi

**Affiliations:** 1 Department of Psychiatry, Interfaith Medical Center, Brooklyn, USA

**Keywords:** glycopyrrolate, nmda, gaba, manic, bipolar disorder, ptyalism, sialorrhea

## Abstract

Lithium is a mood stabilizer frequently used in psychiatry to treat bipolar disorder. Because lithium has a narrow therapeutic index, it requires frequent monitoring for its toxicity. Lithium toxicity requires monitoring of serum lithium and clinical assessment by clinicians. Sialorrhea, also known as excessive drooling, hypersalivation, or ptyalism, is common among psychiatric patients. Sialorrhea, an infrequent and embarrassing side effect of lithium, has been reported at varying serum levels, either at subtherapeutic or in the normal range. Here, we present the case of a patient with sialorrhea associated with oral lithium therapy at the subtherapeutic serum level.

## Introduction

Lithium is a commonly used mood stabilizer for treating bipolar disorder [1]. This psychotropic agent has been studied extensively since its introduction in psychiatry for over 60 years. Lithium is effective in the management of acute manic and depressive episodes, as well as in decreasing the recurrence of mood episodes and reducing the risk of suicidal behaviors [1,2]. The mechanisms by which lithium exerts its therapeutic effects are still not fully understood [3,4]. Lithium exerts its neuroprotective effects at the cellular level by inhibiting two enzymatic pathways, namely, inositol mono-phosphatase (IMPase) within the phosphatidylinositol signaling pathway only when they are in excess, and, second, the G-coupled receptor pathway via inhibition of protein kinase C-glycogen synthase kinase 3 (PKC-GSK3) and its downstream target, myristoylated alanine-rich C (MARCKS), thus deriving its antimanic effects [1,4,5]. It also decreases excitatory neurotransmission at the cellular level by modulating neurotransmitter (dopamine and glutamate) levels [2]. It increases inhibitory transmission by increasing GABA and serotonin levels [6]. Lithium also downregulates N-methyl D-aspartate (NMDA) receptors [7]. It is excreted almost entirely (95%) by the kidney, with only a tiny amount lost with feces and sweat [8].

Common side effects of lithium treatment include dyspepsia, nausea, vomiting, and diarrhea. Upon initiation of treatment, patients have also complained of tremors, weight gain, increased thirst, polyuria, and acne [3].

Sialorrhea, also known as excessive drooling, hypersalivation, or ptyalism, is common among psychiatric patients [9]. It has been implicated as a side effect of many psychotropic agents, for example, clozapine and olanzapine [9]. Other pathologies that can contribute to this phenomenon include postural dysfunction, saliva hypersecretion, dental malocclusion, and salivary spill recognition, especially in patients with neurologic illnesses [10]. Lithium-induced side effects may cause a range of physical and psychosocial complications. Examples include social stigmatization, perioral chapping, aspiration, dehydration, skin breakdown, bad odor, and infection, distressing patients and their families and negatively influencing their quality of life [11]. For the patient with an intact sensory awareness of saliva, it can present with significant embarrassment and isolation, leading to social stigma [12]. Regular evacuation or occult drooling into a tissue or spit cup is socially embarrassing and debilitating, resulting in isolation, social stigma, and low self-esteem [12]. Hypersalivation is a rare side effect of lithium. There is no widely reported literature on sialorrhea related to lithium use.

## Case presentation

A 29-year-old female of South Korean descent, single, unemployed, supported by welfare, domiciled, had past psychiatric histories of bipolar disorder and generalized anxiety disorder, but no significant past medical history. The patient was brought to the psychiatry emergency department (ED) by the emergency medical services (EMS) activated by her brother due to disorganized and aggressive behavior in the context of medication non-compliance.

On assessment in the ED, the patient was floridly manic. She reported a euphoric mood, sleeping only two to three hours per night over the past three days leading to her presentation.

She reported spending a lot of money recently on items she did not need. She endorsed an erotomanic delusion of being in love with a South Korean male model on Instagram with whom she communicated telepathically. Her speech was rapid and pressured. She was hyperactive and pacing the emergency room. The patient had five previous hospitalizations for manic episodes since 2016, with the most recent hospitalization a few months before the current presentation. Past medication trials included lithium, aripiprazole, and lumateperone. She was not compliant with her prescribed medications and outpatient follow-up appointments. The patient had poor insight, poor judgment, and poor impulse control. She was judged a danger to herself and others at the time and was deemed not to be psychiatrically stable, requiring acute inpatient hospitalization for stabilization.

During her medical workup, the patient complained of a mild cough and tested positive for coronavirus disease 2019 (COVID-19) but was otherwise asymptomatic. She was transferred to the medical floor for monitoring of her symptoms and medical stabilization. Her vital signs were stable, and her oxygen saturation was 98% on room air. Complete metabolic panel and other laboratory tests were within the normal range. Chest X-ray was normal, electrocardiography showed no abnormalities, and her baseline lithium level was in the subtherapeutic range at <0.3 mEq/L (normal serum level = 0.6-1.2 mEq/L).

The infectious disease team was consulted, and they started her on a five-day course of Paxlovid for COVID-19 treatment. She was started on an oral dose of 300 mg lithium twice daily for mood stabilization and 3 mg of oral melatonin for insomnia.

While on the medical floor, the patient was followed up by the consultation and liaison psychiatric team, which recommended continuation of lithium at 300 mg twice daily oral dose for mood stabilization, clonazepam 0.5 mg twice daily oral dose for anxiety, and 50 mg of oral diphenhydramine at night as needed for insomnia.

Her repeat COVID-19 polymerase chain reaction test was negative, but her serum sodium level on day five was 132, which was repleted. The patient was deemed medically stable on day nine, and the consultation and liaison team recommended up-titration from 300 mg oral lithium twice daily to 300 mg thrice daily. Her lithium level measured after five days was still subtherapeutic at 0.3 mEq/L (normal serum level = 0.6-1.2 mEq/L).

The patient was transferred to the psychiatry unit on day 10. On arrival at the psychiatric unit, the patient reported excessive drooling and tremors. The oral examination was unremarkable. The patient had no sores on the side of the oral cavities indicative of angular cheilitis and no gastric reflux or ulcer symptoms. The patient denied ever having sialorrhea, either from antipsychotics or lithium use. The patient was educated on the possible cause of her sialorrhea and treatment options. The patient was given psycho-education regarding possible treatment options for sialorrhea, including discontinuation of lithium. The patient verbalized understanding and agreed to it.

The patient was treated with 0.1 mg glycopyrrolate intramuscular injection once. Excessive salivation decreased following treatment, but an oral glycopyrrolate tablet of 1 mg daily was continued for eight days to prevent repeat sialorrhea. The patient was started on benztropine 1 mg per oral twice daily for extrapyramidal symptoms.

Lithium was discontinued on day 12 according to the preference of the psychiatrist, and the extrapyramidal symptoms began to resolve a few days following the discontinuation of the lithium. There was an eventual complete resolution of symptoms. The patient was then started on quetiapine 25 mg oral dose at bedtime and oxcarbazepine 300 mg orally twice daily for mood stabilization. There was a good resolution of her manic symptoms, but the erotomanic delusions continued. She was discharged after 14 days in patient management with an aftercare appointment for outpatient follow-up.

## Discussion

Sialorrhea can occur not only at toxic serum levels (1.5 mEq/L and above) but also below toxic serum levels [5,13,14]. Our case report highlights sialorrhea occurring at a serum level of <0.3 mEq/L, below the therapeutic range (therapeutic range = 0.6-1.2 mEq/L) and toxic serum level (1.5 mEq/L and above). Other studies have highlighted sialorrhea occurring at serum levels of 0.85 mEq/L [14] and 0.9 mEq/L [5]. In both cases, the levels were below the serum level of lithium toxicity [5,14]. Hence, clinicians should be clinically vigilant of this side effect.

Sialorrhea, a rare side effect of lithium, has been reported at various time courses of lithium therapy. In the case report by Halder et al. [10], sialorrhea began after one month of therapy initiation. In the case report by Bou Khalil et al. [5], it occurred within three days of lithium therapy initiation. In the case report by Donaldson [14], sialorrhea occurred 10 days after the therapy initiation. In our case report, sialorrhea occurred after 10 days of lithium therapy initiation, which is similar to the case report by Donaldson [14]. These reports signify that the duration between initiating lithium therapy and sialorrhea initiation does not follow a regular time interval pattern.

Reports suggest that lithium levels in the serum are directly related to the severity of hypersalivation, i.e., the quantity or volume of hypersalivation increases with serum levels [15]. Lithium levels affect the catecholamine metabolism in the central nervous system but have no effects on the peripheral nervous system [15,16]. Another postulation is the direct effect of lithium on salivary gland secretion [15]. The secretion of lithium ions into the saliva causes constant, localized irritation [15,16], triggers the central chemoreceptors in the emetic zone, and produces a transient rise in urinary aldosterone [15].

Drooling can be caused by an increased salivary flow that is not compensated for by swallowing or impaired swallowing that cannot handle the reduced or normal salivation. Our patient’s physical examination did not reveal intractable, localized irritation, she never complained of nausea or dysgeusia, and she had no other signs of lithium toxicity. Therefore, organic causes of hypersalivation and lithium toxicity were ruled out, as our patient’s lithium level was below the toxic serum range.

In previous case reports, the addition of anticholinergic medications caused a reduction in sialorrhea [10,15], and a reduction in the lithium dose resulted in the remission of hypersalivation [13]. In another report, stopping lithium therapy was necessary to achieve remission of excessive salivation [5]. In our case, similar to that reported by Donaldson [14], adding an anticholinergic agent in 1 mg of glycopyrrolate provided symptomatic relief and did not result in the return of symptoms. However, stopping lithium therapy was clinically imperative to achieve remission.

## Conclusions

Sialorrhea resulting from lithium therapy is rare. It is an embarrassing side effect that negatively affects social, occupational, and personal functioning. This can negatively affect the patient’s self-esteem and lead to social isolation and medication non-compliance. Clinicians should be vigilant to recognize this rare but significant side effect of lithium therapy. At the presentation of a patient with sialorrhea, clinicians should review the patient’s serum lithium levels and monitor for other signs and symptoms of lithium toxicity. There are reported cases where the serum lithium was subtherapeutic, within normal range, and might or might not be associated with clinical signs of lithium toxicity. However, clinicians should be aware that while presenting with hypersalivation, the patient’s serum levels may be below, within normal, or in the toxic range. Consequently, treatment should be planned accordingly. Both dose reduction and the addition of anticholinergic agents can significantly improve sialorrhea induced by lithium therapy.
